# Targeting cell cycle regulators in hematologic malignancies

**DOI:** 10.3389/fcell.2015.00016

**Published:** 2015-04-09

**Authors:** Eiman Aleem, Robert J. Arceci

**Affiliations:** ^1^Department of Child Health, The Ronald A. Matricaria Institute of Molecular Medicine at Phoenix Children's Hospital, University of Arizona College of Medicine-PhoenixPhoenix, AZ, USA; ^2^Department of Zoology, Faculty of Science, Alexandria UniversityAlexandria, Egypt

**Keywords:** Cdk6, Cdk1, Wee-1, Cyclin C, Cdk8, Cdk19, leukemia, mouse models

## Abstract

Hematologic malignancies represent the fourth most frequently diagnosed cancer in economically developed countries. In hematologic malignancies normal hematopoiesis is interrupted by uncontrolled growth of a genetically altered stem or progenitor cell (HSPC) that maintains its ability of self-renewal. Cyclin-dependent kinases (CDKs) not only regulate the mammalian cell cycle, but also influence other vital cellular processes, such as stem cell renewal, differentiation, transcription, epigenetic regulation, apoptosis, and DNA repair. Chromosomal translocations, amplification, overexpression and altered CDK activities have been described in different types of human cancer, which have made them attractive targets for pharmacological inhibition. Mouse models deficient for one or more CDKs have significantly contributed to our current understanding of the physiological functions of CDKs, as well as their roles in human cancer. The present review focuses on selected cell cycle kinases with recent emerging key functions in hematopoiesis and in hematopoietic malignancies, such as CDK6 and its role in MLL-rearranged leukemia and acute lymphocytic leukemia, CDK1 and its regulator WEE-1 in acute myeloid leukemia (AML), and cyclin C/CDK8/CDK19 complexes in T-cell acute lymphocytic leukemia. The knowledge gained from gene knockout experiments in mice of these kinases is also summarized. An overview of compounds targeting these kinases, which are currently in clinical development in various solid tumors and hematopoietic malignances, is presented. These include the CDK4/CDK6 inhibitors (palbociclib, LEE011, LY2835219), pan-CDK inhibitors that target CDK1 (dinaciclib, flavopiridol, AT7519, TG02, P276-00, terampeprocol and RGB 286638) as well as the WEE-1 kinase inhibitor, MK-1775. The advantage of combination therapy of cell cycle inhibitors with conventional chemotherapeutic agents used in the treatment of AML, such as cytarabine, is discussed.

## Introduction

Hematologic malignancies account for around 9% of all cancers and represent the fourth most frequently diagnosed cancer in both men and women in economically developed regions of the world (Smith et al., 2011). Around 50% of the children with cancer, younger than 15, have leukemia or lymphoma and acute myeloid leukemia (AML) is responsible for approximately half of the leukemic deaths in children (Meshinchi and Arceci, 2007).

Normal hematopoiesis is dependent upon tightly controlled and inter-related mechanisms regulating cell proliferation and differentiation. Hematopoiesis is initiated in the bone marrow (BM) by multipotent hematopoietic stem cells (HSC). The HSC population is relatively quiescent (Bradford et al., 1997; Cheshier et al., 1999), but upon cell cycle entry it will give rise to differentiating progenitor populations that undergo massive proliferative expansion in order to replenish the blood system on a daily basis but also in response to stress, such as injury or infection (Weissman, 2000). Quiescence is thought to prevent stem cell exhaustion (Orford and Scadden, 2008) and to help protect stem cells from acquiring mutations leading to their malignant transformation (Park and Gerson, 2005; Wang and Dick, 2005; Lobo et al., 2007). Therefore, the balance between proliferation and quiescence in HSCs must be carefully regulated to ensure homeostasis (Wilson et al., 2008). Cell cycle regulators play a crucial role in controlling these processes and have been previously reviewed (Passegue et al., 2005; Pietras et al., 2011; Tesio and Trumpp, 2011; Cabezas-Wallscheid et al., 2014). The current review will focus on the impact of such cell cycle regulators on hematological-derived cancers and their potential as therapeutic targets.

## Acute myeloid leukemia from mutations to dysregulated pathways leading to aberrant cell proliferation

Approximately 300 genes have been reported to be altered in hematologic malignancies (Catalog of Somatic Mutations in Cancer at the Wellcome Trust Sanger institute). In most hematologic malignancies, normal hematopoiesis is interrupted by uncontrolled growth of a genetically altered stem or progenitor cell. AML, which accounts for 75% of acute leukemia, is a complex and heterogeneous disease (Appelbaum et al., 2007). In AML, a HSC or progenitor cell will be transformed to a leukemia stem cell (LSC) through accumulation of genomic alterations that enable the LSC to maintain its ability of self-renewal but without normal terminal differentiation (Renneville et al., 2008).

Several lines of evidence have led to a model that AML arises from the cooperation between two classes of genetic alterations (Gilliland et al., 2004) in which *class I mutations* confer a proliferation or survival advantage to blast cells. These typically include genes encoding key proliferative tyrosine kinase receptors, such as the FMS-like tyrosine kinase 3 (*FLT3*) and *c*-*KIT*, as well as down-stream signaling regulators, such as the *RAS* oncogene and *PTPN11* (protein tyrosine standard phosphatase non-receptor 11) gene that encodes SHP-2. Other mutated genes in this category include *JAK2* and the *IL-3* receptor family. *Class II mutations* that impair myeloid differentiation typically include mutations in the *AML1* (*CBFA2 or RUNX1*), *CEBPA, WT1* and *PU.1* (Renneville et al., 2008; Arceci and Aplenc, 2009; Pui et al., 2011; Schnerch et al., 2012). The interplay of dysregulated proliferative and differentiation pathways in turn have important consequences for the altered regulation of the cell cycle controllers, such as cyclins, cyclin-dependent kinases (CDKs), checkpoint kinases, and mitotic kinases.

Furthermore, mutations in genes regulating chromatin and/or methylation states in hematopoietic progenitors are emerging as critical events in AML and have prognostic importance. These genes include the tet methylcytosine dioxygenase 2 (*TET2*), mutated in 7–25% of AML, the isocitrate dehydrogenase 1 (*IDH1, IDH2*), mutated in 15–30% of AML and the DNA methyltransferase 3A (*DNMT3A*), mutated in 15–25% of AML (Shih et al., 2012; Conway O'brien et al., 2014; Greenblatt and Nimer, 2014).

Targeted cancer therapies are developed to interrupt a specific component of the complex network of altered signaling pathways that ultimately results in uncontrolled cell proliferation. However, there is a significant cross talk between these signaling pathways and the same pathway is usually activated by multiple receptors. Therefore, if the function of one protein located upstream the signaling pathway is inhibited another protein will most likely compensate the interrupted function. The outcome will still be uncontrolled cell proliferation. However, most of these pathways converge in a signal that activates the cell cycle, which consists of a series of well-defined, unidirectional sequence of events from G1 to M. Therefore, blocking one phase of the cell cycle should theoretically interrupt the remaining phases and halt proliferation.

The present review focuses on the alterations and therapeutic targeting of selected downstream cell cycle kinases based on their emerging key functions in hematopoiesis and in the development and progression of hematopoietic malignancies. A brief overview of CDKs and their multifaceted biological functions is first presented followed by the rationale for their therapeutic targeting. Although human tumors are more genetically complex than mouse tumors, genetically engineered mouse models have significantly contributed to our understanding of CDK functions in normal development and remain indispensable for understanding the development and progression of human cancer, as well as response to treatment. For each of the following cell cycle kinases: CDK6, CDK1, WEE-1 and cyclin C/CDK8/CDK19 we will present (1) an overview of the current knowledge of their biological functions gained from knockout mouse models, (2) the role of each protein in human cancer with emphasis on hematologic malignancies, and (3) the pharmacological inhibitors currently in clinical development to target these kinases in hematologic malignancies.

## Cyclin-dependent kinases

In order for a cell to undergo successful division, it has to perform four key tasks in a highly ordered fashion. First, there is a preparatory synthetic phase (G1) that results in an increased cell size in anticipation of DNA replication (S phase). Cells then proceed through (G2-phase) to prepare to equally segregate duplicated DNA (M phase) and finally divide into two equal daughter cells. From G1 a cell can also exit the cell cycle and enter a state of quiescence (G0), undergo differentiation or re-enter the cell cycle to proliferate in response to mitogenic signals.

**Figure 1:**
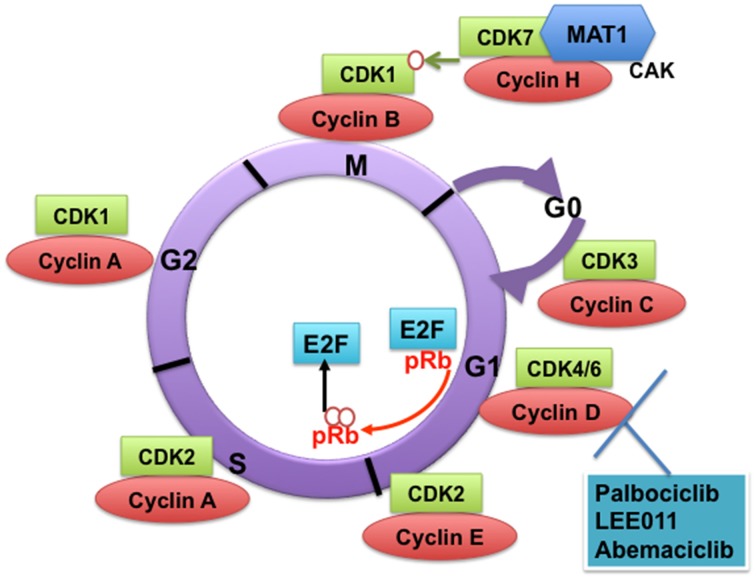
**Cyclin-dependent kinases (CDKs) and their cyclin regulatory subunits**. CDK-cyclin complexes with direct functions in regulating the cell cycle. CDK3/cyclin C drives cell cycle entry from G0. CDK4/6/cyclin D complexes initiate phosphorylation of the retinoblastoma protein (pRb) and they sequester p21^Cip1^ and p27^kip1^ (not shown), which are both inhibitors of CDK2, thus promoting the activation of CDK2/cyclin E complex. In late G1, CDK2/cyclin E complex completes phosphorylation and inactivation of pRb, which releases the E2F transcription factors and G1/S transition takes place. DNA replication takes place in S phase. CDK2/cyclin A complex regulates progression through S phase and CDK1/cyclin A complex through G2 phase in preparation for mitosis (M). Mitosis is initiated by CDK1/cyclin B complex. The activity of CDK1/cyclin B is tightly regulated by activating phosphorylation by the CDK-activating kinase CAK (a heterodimer of cyclin H-CDK7-MAT1) and inhibitory phosphorylations by Wee1 and Myt1 on Tyr15 and Thr14 (not shown). The specific CDK4/CDK6 pharmacological inhibitors described in this study are shown.

The core molecular machinery controlling the mammalian cell cycle consists of a family of serine/threonine protein kinases called cyclin-dependent kinases (CDKs). These are catalytic subunits, which are activated in most cases by association with cyclin regulatory subunits. The activity of CDK/cyclin complexes is further regulated by CDK-inhibitors (CKIs), phosphorylation and dephosphorylation, ubiquitin-mediated degradation, transcriptional regulation, substrate recognition, and subcellular localization (Aleem and Kaldis, 2006). The family of CDKs/cyclins/CKIs contains more than 30 members (Figure 1; Supplementary Table 1) and they are implicated in essential cellular functions such as transcription, DNA damage repair, epigenetic regulation, metabolism, proteolytic degradation, stem cell self renewal, neuronal functions, and spermatogenesis (Lim and Kaldis, 2013).

## CDKs with direct functions in cell cycle regulation

The classical CDKs that directly regulate the mammalian cell cycle in complexes with cyclin subunits include CDK3, CDK4, CDK6, CDK2, and CDK1. CDK3 promotes cell cycle entry from quiescence in association with cyclin C (Ren and Rollins, 2004). CDK8 has also been suggested to play a role in cell cycle entry from G0 and in the G1/S transition (Szilagyi and Gustafsson, 2013). In its simplest model, the mammalian cell cycle proceeds as follows: In early G1, CDK4/CDK6 in complex with cyclin D receive mitogenic signals that result in activation of cell cycle entry (Figure 1). Key signaling events include initiation of retinoblastoma protein (pRb) phosphorylation and the sequestration of p21^Cip1^ and p27^kip1^, which are both inhibitors of CDK2, thus promoting the activation of CDK2/ cyclin E complex (Sherr and Roberts, 1999). In late G1, CDK2 in complex with cyclin E completes the phosphorylation and hence inactivation of pRb, which in turn releases the E2F transcription factors (Kaldis and Aleem, 2005). E2F promotes transcription of cyclin E that is necessary for the G1/S transition.

Progression through S phase is mediated by CDK2/cyclin A complex. Mitosis is then initiated by CDK1/cyclin B complexes. CDK1/cyclin A complexes contribute to the preparation for mitosis in G2 phase (Nigg, 1995; Edgar and Lehner, 1996). The activity of CDK1/cyclin B is tightly regulated by activating phosphorylation by the CDK-activating kinase (CAK) (a heterodimer of cyclin H and CDK7) and inhibitory phosphorylations by WEE-1 and Myt1 on Tyr15 and Thr14. Mitosis starts after WEE-1 is degraded and CDC25C phosphatase releases the inhibitory phosphorylation on CDK1/cyclin B.

## CDKs with transcriptional and other functions

In addition to their direct role in the mitotic cell cycle regulation, some classical CDK/cyclin complexes have essential functions in meiosis, such as CDK2 (Berthet et al., 2003; Ortega et al., 2003), in transcription and/or DNA repair (e.g., CDK1, CDK2, CDK6) (Satyanarayana and Kaldis, 2009; Neganova et al., 2011; Wohlbold et al., 2012; Kollmann et al., 2013; Lim and Kaldis, 2013; Scheicher et al., 2015). Other CDKs (referred to in Supplementary Table 1 as non-classical CDKs) act by activating the classical CDKs, such as CDK7/cyclin H (CAK) and the related CDK20, also known as cell cycle related kinase (CCRK) (Wohlbold et al., 2006). Some CDKs function mainly in influencing transcription by phosphorylating the carboxy-terminal domain (CTD) of ribonucleic acid (RNA) polymerase II (RNA pol II) (Bose et al., 2013; Lim and Kaldis, 2013) (Supplementary Table 1). This phosphorylation also serves as a platform for RNA processing and chromatin regulation (Hirose and Ohkuma, 2007).

CDKs that have important transcriptional roles include CDK7/cyclin H/MAT1 complex, a component of the basal transcription factor, TFIIH and facilitates transcriptional initiation (Shiekhattar et al., 1995). CDK8/cyclin C, in addition to its role in transcription (Gonzalez et al., 2014), is also involved in the Wnt/β-catenin pathway (Firestein et al., 2008) and in inhibition of lipogenesis (Zhao et al., 2012). Cyclin C can recruit CDK8 or CDK19 to the CDK8 module of the Mediator complex, which can function as a positive or negative regulator of transcription by RNA pol II (Li et al., 2014; Trakala and Malumbres, 2014). CDK3/cyclin C also plays a role in NHEJ-mediated DNA damage repair (Tomashevski et al., 2010). While CDK9 in complex with cyclin T forms the phospho-transcription elongation factor b (p-TEFb) and promotes transcriptional elongation (Shapiro, 2006), CDK9 also functions in the DNA damage response when complexed with cyclin K (Yu et al., 2010). CDK10/cyclin M phosphorylates the Ets2 transcription factor and positively controls its degradation by the proteasome. Ets2 plays key roles in cancer and development (Guen et al., 2013). CDK11/cyclin L controls the assembly of the RNA pol II mediator complex (Drogat et al., 2012). CDK12 and CDK13 in complex with cyclin K control RNA pol II transcription (Bartkowiak et al., 2010; Blazek et al., 2011; Cheng et al., 2012), and CDK12/cyclin K controls DNA damage response (Blazek et al., 2011). These functions are summarized in Supplementary Table 1.

CDKs with additional functions include CDK5, which plays an important role in controlling neural development and postsynaptic signal integration (Kim and Ryan, 2010), and influences epigenetic regulation through its interaction with Dnmt1 (Lavoie and St-Pierre, 2011). CDK5 also regulates degranulation in human eosinophils (Odemuyiwa et al., 2015). CDK5 plays a role in diabetes; it phosphorylates the peroxisome proliferator-activated receptor γ (PPARγ) at Ser 273, thus stimulating diabetogenic gene expression in adipose tissue (Choi et al., 2010). CDK5 also suppresses ERK2 through direct phosphorylation (Banks et al., 2015). CDK14/cyclin Y is involved in the Wnt/β-catenin pathway (Davidson et al., 2009). CDK15 was reported to attenuate Tumor necrosis factor-related apoptosis-inducing ligand (TRAIL)-induced apoptosis by phosphorylation of survivin (Park et al., 2014). CDK16 in complex with cyclin Y regulates spermatogenesis (Mikolcevic et al., 2012). CDK17 and CDK18 are not well-characterized but a recent report demonstrated that CDK18 is phosphorylated and activated by cyclin A2 and cAMP-dependent protein kinase (PKA) and its knockdown induced polymerized actin accumulation in peripheral areas and cofilin phosphorylation (Matsuda et al., 2014). Furthermore, CDK18 is highly expressed in diabetic human pancreatic islets (Taneera et al., 2013). CDK20 plays a role in ciliogenesis (Ko et al., 2010; Phirke et al., 2011; Yang et al., 2013a)(Supplementary Table 1).

Because of the transcriptional role played by multiple CDKs, pan CDK inhibitors, such as flavopiridol, not only arrest the cell cycle but can interfere with cellular transcription and reduce the expression of short-lived mRNAs and proteins, which include cell cycle regulators and survival factors, such as Mcl-1, thus promoting cell death (Bruyere and Meijer, 2013).

## Rationale for therapeutically targeting CDKs in cancer

The activity of CDK/cyclin complexes is frequently perturbed in human cancer (Lapenna and Giordano, 2009; Malumbres and Barbacid, 2009). Some CDKs can be considered oncogenic, e.g., CDK4 in familial melanoma (Zhao et al., 2012), CDK6 in MLL-rearranged leukemia (Placke et al., 2014), and CDK8 in colon cancer (Firestein et al., 2008). Alteration of CDK-encoding genes or their partner cyclins can lead to their overexpression, amplification and translocation, e.g., cyclin D1 alterations are observed in chronic lymphocytic leukemia (CLL), B-cell acute lymphocytic leukemia (B-ALL), mantle cell lymphoma (MCL), and multiple myeloma (MM). Cyclin D2 translocation and overexpression are described in non-Hodgkins' lymphoma (NHL), CLL, B cell lymphocytic leukemia (BLL) and lymphoplasmacytic lymphomas (LPL). Cyclin D3 translocations are observed in MM and in diffuse large B cell lymphomas (DLBCL) (Supplementary Table 2). In addition to genetic changes, CDK hyperactivation may result from overexpression of positive regulators such as cyclins or the inactivation of their inhibitors, from alternative splicing and expression of truncated cyclin variants or abnormal localization (Peyressatre et al., 2015). For example, CDK2 has not been found mutated in human cancer (Malumbres and Barbacid, 2009). However, its inhibition significantly reduced tumor growth of trastuzumab-resistant xenografts from patients with Her+/amplified cyclin E breast cancer (Scaltriti et al., 2011). Similarly, alterations in the expression and activity of CDK1 have been observed in breast (Kim et al., 2008), gliomas (Chen et al., 2008), lung (Zhang et al., 2011), and colon cancer (Zeestraten et al., 2012). Although the total CDK1 level is high in aggressive NSCLC, its subcellular localization is an important factor because loss of cytoplasmic CDK1 is associated with a poor prognosis (Zhang et al., 2011). Aberrations in transcriptional CDKs with direct modulation of target genes have also been described in human cancer such as CDK8, CDK9/cyclin T1, CDK10, and CDK11 (Peyressatre et al., 2015).

The pharmacological inhibition of CDKs typically results in cell cycle arrest, apoptosis, and transcriptional repression. Currently, inhibitors targeting the transcriptional CDKs such as CDK7 and CDK9 are in preclinical development (Kwiatkowski et al., 2014; Walsby et al., 2014). Interestingly, in mouse models the kinase activities of some cyclin/CDK complexes, such as CDK4 and CDK2, appear dispensable for normal mouse development, but are required for mammary tumor development (Yu et al., 2006; Ray et al., 2011). This provides a possibly selective targeting of tumor cells with potential, relative sparing of normal cells.

## Targeting CDKs in hematologic malignancies

### CDK4/CDK6-cyclin D

CDK4 and CDK6 both bind the D-type cyclins and their kinase activities are inhibited by the INK4 (Inhibitors of CDK4) family of CDK inhibitors, which includes p16^INK4a^. p16^INK4a^ is the most frequently deleted locus in human cancer (Beroukhim et al., 2010); its loss of function can result in a constitutively active CDK4/6, thus driving uncontrolled proliferation. Pharmacological inhibitors of CDK4/6 have been developed and are currently being tested in clinical trials (Supplementary Table 3).

### Mouse models reveal unique roles for CDK6 in hematopoiesis

CDK4 and CDK6 are highly homologous enzymes with basically indistinguishable biochemical properties and play a redundant role during G1/S transition. However, they are required by different tissues during mouse development and their deregulation is associated with different types of human cancer. While CDK4 is required by the pancreatic beta cells and pituitary lactotrophs in mice (Rane et al., 1999; Tsutsui et al., 1999; Moons et al., 2002; Martin et al., 2003; Jirawatnotai et al., 2004), CDK6 has a unique role in hematopoiesis (Malumbres et al., 2004). CDK6 is predominantly expressed in hematopoietic cell types (Della Ragione et al., 1997; Chilosi et al., 1998) and loss of CDK6 in mice causes thymic atrophy and reduction in red blood cells, granulocytes, macrophages, neutrophils and platelets, as well as delayed G1 progression in lymphocytes of *Cdk6*^−/−^ mice, (Malumbres et al., 2004). Moreover, Notch-dependent initiation of T-cell commitment by BM progenitors requires CDK6 (Hu et al., 2009). CDK6 is also a key downstream target in the AKT pathway and *Cdk6*^−/−^ mice are resistant to AKT-driven lymphomagenesis (Hu et al., 2009).

### CDK6 in human ALL, T-LBL, and lymphoma

CDK6 is highly expressed in human T-cell lymphoblastic lymphoma/leukemia (T-LBL/ALL) (Chilosi et al., 1998; Lien et al., 2000; Schwartz et al., 2006; Nagel et al., 2008). CDK6 is also a target of the tumor suppressor microRNA *hsa-mir-124a*, which is frequently hypermethylated and downregulated in ALL (Agirre et al., 2009). Epigenetic downregulation of *mir-124a in vitro* and *in vivo* leads to overexpression of CDK6 and increased proliferation of ALL cells that is in turn decreased by the CDK6 inhibitor palbociclib (PD0332991) (Agirre et al., 2009). In this study, patients with ALL with methylation of *hsa-miR-124a* showed a significant up-regulation of CDK6 expression compared with non-methylated patients. Moreover, patients in the methylated group had a significantly higher relapse and mortality rates as well (Agirre et al., 2009). Hypermethylation of *hsa-miR-124a* was also found to be an independent prognostic factor for disease-free survival (DFS) and overall survival (OS) in patients with ALL. Based on this, targeting CDK6 was suggested as a potential therapeutic strategy for patients with ALL (Agirre et al., 2009).

Chromosomal translocations of CDK6 in patients suffering from B-lymphoid cancers have been documented as well (Brito-Babapulle et al., 2002). Although CDK4/CDK6 pharmacological inhibitors were developed to target their kinase activity as the cause for deregulated proliferation in cancer, additional important cancer-relevant CDK6 kinase-independent functions have been recently discovered in B-cell receptor (BCR)-ABL transformed B-cell leukemia/lymphoma cells (Kollmann et al., 2013). For instance, CDK6 overexpression has been reported to have an anti-proliferative effect *in vivo* resulting in the delay of tumor formation. The seemingly contradictory result may be due to the ability of CDK6 to induce the expression of its inhibitor, the tumor suppressor p16^INK4a^, which then arrests the cell cycle. However, when p16^INK4a^ is inactivated by promoter methylation in a mouse model of T-cell lymphoma, CDK6 acts as an oncogene, as expected. Therefore, induction of p16^INK4a^ by CDK6 protects cells from high CDK6 activity through a negative feedback loop (Otto and Sicinski, 2013). In transformed lymphoid cells, the relative amounts of the two proteins define the rate at which tumor progression occurs. This may also reflect the situation in human lymphoid malignancies, where CDK6 and p16^INK4a^ are expressed in an inverse manner (Kollmann and Sexl, 2013). In addition to the induction of p16^INK4a^, CDK6 also induces transcription of vascular endothelial growth factor A (VEGFA), a known angiogenic factor and tumor promoter, thereby linking two hallmark cancer features (Kollmann and Sexl, 2013; Otto and Sicinski, 2013). CDK6 binds the promoters of p16^INK4a^ and VEGF-A through interaction with the transcription factors STAT-3 and c-Jun (Kollmann et al., 2013). Thus, CDK4/6 inhibitors may have a dual effect in the types of malignancies with p16^INK4a^ deletion, as they will target both proliferation and angiogenesis.

### CDK6 is a driver and therapeutic target in MLL-rearranged AML, in infant ALL and in FLT3-ITD driven AML

The mixed lineage leukemia (*MLL*) gene, located on chromosome 11q23, has important roles in epigenetic patterning in leukemia (Jin et al., 2010). Chromosomal rearrangements involving the *MLL* gene are found in 5–10% of pediatric and adult AML (Antony-Debre and Steidl, 2014), and in 80% or greater in cases of infant ALL (Van Der Linden et al., 2014); these translocations are usually associated with a poor prognosis. t(11q23) results in fusion of the *MLL* gene to a broad spectrum of partner genes (Meyer et al., 2013). A key functional feature of (*MLL*) rearrangements is their ability to confer leukemia-initiating activity to HSPC (Krivtsov et al., 2008). Recently, CDK6 was found to be a direct target of MLL-fusion proteins in MLL-AF9-driven AML (Placke et al., 2014) and of infant MLL-AF4-driven ALL (Van Der Linden et al., 2014), resulting in the transcriptional activation of CDK6. This was shown in cell lines, a murine model of MLL-AF9-driven AML, and in infant ALL patient samples. A likely key mechanism by which CDK6 drives MLL-AF9-driven AML is through inhibition of myeloid differentiation (Placke et al., 2014). An earlier report also identified CDK6 as a target of *microRNA-29a*, which regulates myeloid differentiation in HSPCs and in AML cell lines (Wang et al., 2013). These studies showed that the catalytic activity of CDK6 is required for this function because expression of a mutant form of CDK6 with disrupted kinase function is unable to block the differentiation induced by shRNA-mediated depletion of CDK6 in MLL-rearranged AML cells (Placke et al., 2014).

About 25% of cases of AML express a constitutively active *FLT3* internal tandem duplication (*FLT3-ITD*), an activating mutant form of the receptor tyrosine kinase FLT3 (Rombouts et al., 2000; Small, 2006). Inhibition of FLT3-ITD in AML leads to down regulation of cyclin D2 and D3, both activators of CDK6 (Wang et al., 2007), which suggests that CDK6 may be an additional candidate for therapeutic targeting in *FLT3-ITD-driven* AML.

### Mouse models of the D-type cyclins reveal essential roles in hematopoiesis

The D-type cyclins, especially D2 and D3, which bind and activate CDK4/CDK6, play unique roles in hematopoiesis, and hematologic malignancies. Mice lacking cyclin D2 show impaired proliferation of B-lymphocytes (Kushner et al., 2005) and *cyclin D3*^−/−^ mice display hypoplastic thymuses and are resistant to Notch-driven leukemia (Sicinska et al., 2003). Moreover, ablation of cyclin D3 in mice bearing Notch1-driven T-cell acute lymphoblastic leukemia (T-ALL) triggers tumor cell apoptosis. Such selective killing of leukemic cells can also be achieved by inhibiting cyclin D-associated kinase activity in mouse and human T-ALL models (Choi et al., 2012). Acute ablation of the 3 D-type cyclins in adult mouse BM revealed that adult HSCs are dependent on D-type cyclins for survival, and their loss results in massive apoptosis of all hematopoietic cell types due to upregulation of the expression of the death receptor Fas and its ligand FasL (Choi et al., 2014).

### Genomic alterations of the D-type cyclins in hematologic malignancies

Chromosomal translocation involving cyclin D1 is a common genetic event in the pathogenesis of B-cell lymphomas, especially MCL (Supplementary Table 2). MCL is characterized by the t(11;14)(q13;q32) translocation that juxtaposes the cyclin D1 gene (*CCND1*) to the immunoglobulin (*Ig*) heavy chain gene (Bertoni et al., 2006), and results in constitutive overexpression of cyclin D1 under the control of an active *Ig* locus (Baker and Reddy, 2012). Single nucleotide polymorphisms are also thought to contribute to increased levels of cyclin D1 via production of an alternatively spliced isoform (Baker and Reddy, 2012). The G/A870 polymorphism is thought to hinder normal splicing at the exon 4/5 boundary resulting in the expression of a truncated cyclin D1 protein (Betticher et al., 1995), cyclin D1b, that forms an active complex with CDK4, (Lu et al., 2003; Solomon et al., 2003) but it lacks the residue necessary for nuclear export and subsequent cytoplasmic degradation (Diehl et al., 1997), thereby stabilizing cyclin D1 protein and increasing its oncogenic potential (Alt et al., 2000). The CCND1 A/A variant has been linked to increased susceptibility (Hou et al., 2005) and a lower probability of event-free survival in children with ALL compared to carriers of the G variant (Costea et al., 2003).

Cyclin D2 protein frequently shows high levels of expression in B cell malignancies, such as BLL, LPL (Delmer et al., 1995) and in CLL (Motokura and Arnold, 1993). Cyclin D2 is essential for BCR-dependent proliferation of B cells (Mohamedali et al., 2003) and for BCR/ABL-dependent transformation of BM cells (Jena et al., 2002). Patients with chronic myeloid leukemia (CML) carry the *BCR*/*ABL* fusion protein (De Klein et al., 1982; Shtivelman et al., 1985). Therefore, modulation of its downstream target cyclin D2-associated activity may be important in CML. The t(6;14)(p21.1;q32.3) translocation that juxtaposes the *CCND3* and *IGH* loci resulting in high-level expression of *CCND3* has been reported in MM (Bergsagel and Kuehl, 2001) and in several subtypes of mature B-cell malignancies including DLBCL (Sonoki et al., 2001).

While there are no inhibitors that directly target cyclin D, alternative therapeutic strategies targeting CDK4/CDK6 could be potentially useful in hematologic malignancies with altered D-type cyclins.

## CDK4/CDK6 inhibitors

### Palbociclib

Palbociclib (PD0332991, Pfizer) is an orally administered small molecule inhibitor of CDK4/6 (Fry et al., 2004) undergoing active clinical trial testing (Supplementary Table 3). As pRb is a substrate of CDK4/6-cyclin D, palbociclib targets Rb+ tumor cells *in vitro*, inducing G1 arrest, with concomitant elimination of phospho-Rb and inhibition of E2F-dependent transcription (Bose et al., 2013).

Because of the critical role played by CDK6 in *MLL*-rearranged AML and ALL, as well as in FLT3-ITD driven AML, CDK6 may be an effective therapeutic target in these leukemias. In AML cell lines bearing the *FLT3-ITD* mutation, PD0332991 as single-agent induces sustained cell cycle arrest and prolonged survival in an *in vivo* model of *FLT3-ITD* AML (Wang et al., 2007). It also causes an initial cell-cycle arrest in well-established *FLT3* wild-type (wt) AML cell lines, but this is overcome by down-regulation of p27^Kip1^ and reactivation of CDK2. This acquired resistance is not observed in *FLT3-ITD* and a *FLT3* wt samples from patients with primary AML (Wang et al., 2007). This study suggests the activation of CDK2 as a potential mechanism of resistance to PD0332991. Therefore, it might be worthwhile to test combined inhibition of CDK4/CDK6 and CDK2 in such cases, while closely monitoring toxicity. Flavopiridol and AT7519 (discussed below) are drugs that target six CDKs including CDK2/4/6 (Bose et al., 2013). Although *in vitro* studies consider CDK2 to be a major player in proliferation and apoptosis in hematopoietic cells, it is not required for proliferation and differentiation of hematopoiesis *in vivo* (Berthet et al., 2007).

In *MLL*-rearranged leukemia, PD0332991 strongly inhibits colony formation in methylcellulose of MLL-AF9 positive AML cell lines and of mononuclear cells from patient derived AML samples (Placke et al., 2014). Furthermore, inhibition of CDK4/6 kinase activity using PD0332991 leads to inhibition of cell cycle progression and accumulation in the G_o_/G_1_cell cycle phase in T-ALL primary samples, triggers apoptosis, and blocks progression of leukemia in a mouse model of T-ALL (Sawai et al., 2012). Preclinical testing has also demonstrated efficacy of PD0332991 in MM *in vitro* and *in vivo* and in MCL (Baughn et al., 2006; Marzec et al., 2006). It potently induces G1 arrest, but not apoptosis, in primary BM myeloma cells *ex vivo*. In addition, PD0332991 markedly enhances the killing of myeloma cells by dexamethasone (Baughn et al., 2006). In the 5T33MM myeloma mouse model, PD0332991 treatment leads to tumor suppression and a significant improvement in survival. Induction of G1 arrest by PD0332991 sensitizes 5T33MM tumor cells to killing by bortezomib (Menu et al., 2008).

The Food and Drug Administration designated palbociclib a breakthrough therapy in April 2013 (Brower, 2014). Currently, there are 36 clinical studies that are either completed or ongoing involving PD0332991 as single agent or in combination with other drugs for the treatment of various types of cancer (clinicaltrials.gov, accessed 11/2014). Seven studies involve hematologic malignancies (Supplementary Table 3) and 29 studies are for advanced solid malignancies. Phase I trials are being done for patients with MM, MCL, acute leukemia (AL), NHL and myelodysplasia (MDS). Two completed phase I studies have established dosing regimens of 200 mg daily for 2 weeks followed by 7 days off treatment (21-day cycles; Schedule 2/1) (Schwartz et al., 2011), or 125 mg daily for 3 weeks of 4 (Flaherty et al., 2012). In each case, neutropenia was the dose-limiting toxicity (DLT). This toxicity profile is probably a result of transient growth arrest in hematopoietic precursor cells (Roberts et al., 2012). Other side effects associated with conventional cytotoxic drugs, such as gastrointestinal toxicity, alopecia, or mucositis, fever or infection, were rare (Dickson, 2014). PD0332991 has also shown promising activity in 17 patients with relapsed MCL. Five patients achieved progression-free survival (PFS) of greater than a year, with 1 complete (CR) and 2 partial responses (PRs) (18% objective response rate) (Leonard et al., 2012).

### LEE011

LEE011 (Novartis) is an orally bioavailable small molecule that inhibits CDK4/6 at nanomolar concentrations (Gelbert et al., 2011; Kim et al., 2013). LEE011 demonstrated antitumor activity in several models. There are currently 16 active clinical trials with LEE011 as single agent or in combination with other drugs. Most of the trials are for solid tumors, including melanoma with BRAF or NRAS mutations, and breast cancer, neuroblastoma, malignant rhabdoid tumors (clinicaltrials.gov, accessed 11/2014). Two trials are in lymphomas (Supplementary Table 3). Results from a phase I study in patients with advanced solid tumors or lymphomas recommended doses of 600 mg daily (continuous) and 900 mg daily for 3 weeks of 4 for subsequent Phase II trial testing. As with palbociclib, neutropenia was the major toxicity (Infante et al., 2013; Dickson, 2014).

### Abemaciclib

Abemaciclib formerly LY2835219 (Eli Lilly), like palbociclib and LEE011 is a small molecule selectively targeting CDK4/CDK6 and is orally available. Preclinical data for abemaciclib show antitumor activity in a number of models, and the drug has also been shown to cross the blood–brain barrier (Sanchez-Martinez et al., 2011). There are 10 ongoing clinical trials using abemaciclib as a single agent or in combination for the treatment of solid tumors, such as breast and lung cancer; as well as for lymphoma and MCL (Supplementary Table 3; clinicaltrials.gov, accessed 11/2014). There are 3 Phase III trials (Stage IV NSCLC with a detectable KRAS mutation, and two trials for breast cancer). The maximum tolerated dose (MTD) as recommended from a Phase I study of abemaciclib in patients with advanced cancer is 200 mg twice daily (Shapiro et al., 2013). The most common adverse effects were diarrhea, fatigue, and neutropenia.

In general, cancers with p16 loss may also be sensitive to CDK4/CDK6 inhibition, with preclinical data supporting this in melanoma and lung cancer among others (Sheppard and Mcarthur, 2013) (Dickson, 2014). In contrast, cancers that lack Rb function may be resistant to CDK4/CDK6 inhibition because the antitumor effect of CDK4/CDK6 inhibition is partly due to downstream Rb phosphorylation. This category of tumors includes those with Rb loss at the gene level (such as Rb) and those with functional inactivation of Rb protein, such as squamous cell carcinomas of the oropharynx, cervix, and genital tract in which the E7 oncogene of HPV16 inactivates Rb (Wiest et al., 2002; Dickson, 2014).

## CDK1

### CDK1 functions

CDK1 drives the cell cycle through G2 and M phases in association with cyclin A and cyclin B, respectively. The phosphorylation of CDK1 on Thr14 (by Myt1 kinase) and Tyr15 (by WEE-1 kinase) (Mcgowan and Russell, 1995) inhibits its activity during the G2 phase of the cell cycle, while its dephosphorylation on the same sites by CDC25C results in its activation during early mitosis (Boutros et al., 2007). Both CDK1 and WEE-1 inhibitors are currently being used in clinical trials for the treatment of hematologic malignancies (below).

Using genetically engineered mouse models it has been demonstrated that CDK1 could drive all phases of the mammalian cell cycle in the absence of CDK2, CDK4, and CDK6 with subsequent phosphorylation of pRB (Santamaria et al., 2007) by binding additionally to cyclin D and E and to the inhibitors p27^kip1^ and p21^Cip1/Waf1^ (Aleem et al., 2005; Martin et al., 2005; Santamaria et al., 2007; Satyanarayana et al., 2008). Liver-specific deletion of CDK1 results in DNA re-replication because of an increase in CDK2/cyclin A2 activity. Moreover, loss of CDK1 in the mouse liver confers resistance against tumorigenesis induced by activated Ras and silencing of p53 (Diril et al., 2012). Furthermore, CDK1 plays a role in Myc-induced transformation (Goga et al., 2007). Cells transformed by Myc undergo apoptosis through down-regulation of the CDK1 target survivin upon treatment with small-molecule CDK1 inhibitors. CDK1 inhibitors also decrease tumor growth and prolong survival in mouse models of Myc-dependent lymphoma and hepatoblastoma (Goga et al., 2007). Similarly, targeting CDK1, but not CDK4/6 or CDK2, is selectively lethal to MYC-dependent human breast cancer cells (Kang et al., 2014). Thus, because there are currently no compounds directly targeting Myc, CDK1 inhibition may be an important therapeutic approach for human malignancies that overexpress MYC.

CDK1 regulates vital cellular processes in addition to cell cycle (Supplementary Table 2) (Lim and Kaldis, 2013). CDK1 functions in FoxM1 and FoxK2 transcription (Chen et al., 2009; Marais et al., 2010), in homologos recombination-mediated DNA damage repair (Huertas et al., 2008; Chen et al., 2011), in embryonic stem cells (ESC) self-renewal through interaction with Oct4 (Li et al., 2012), and in neural stem cell (NSC) self-renewal through inhibition of Ngn2 (Ali et al., 2011). Furthermore, CDK1 plays a role in epigenetic gene silencing and in mesenchymal stem cell (MSC) differentiation. CDK1 and CDK2 phosphorylate the enhancer of zeste homolog 2 (EZH2), a Polycomb group (PcG) protein, at Thr 350 (Chen et al., 2010). EZH2 is a histone lysine methyltransferase that catalyzes the addition of methyl groups to histone H3 at Lys 27 (H3K27) in target gene promoters, leading to epigenetic silencing (Wei et al., 2011). EZH2 is the catalytic subunit of Polycomb repressive complex 2 (PRC2), which includes SUZ12 (suppressor of zeste 12) and EED (embryonic ectoderm development) (Mrozek et al., 2001; Tenen, 2003). It enhances cancer-cell invasiveness and regulates stem cell differentiation (Wei et al., 2011). Phosphorylation of EZH2 by CDK1 and CDK2 at Thr350 is important for recruitment of EZH2 and maintenance of H3K27me3 levels at EZH2-target loci, thus resulting in epigenetic silencing (Chen et al., 2010). Activation of CDK1 also promotes MSC differentiation into osteoblasts through phosphorylation of EZH2 at Thr 487. This phosphorylation disrupts EZH2 binding with the other PRC2 components SUZ12 and EED, and inhibits EZH2 methyltransferase activity, resulting in inhibition of cancer-cell invasion as well (Wei et al., 2011).

### CDK1 in hematologic malignancies

It has been reported that CDK1 plays an important role in AML (Biggs et al., 2006; Zhang et al., 2008; Radomska et al., 2012; Hedblom et al., 2013) and in chronic myelomonocytic leukemia (CMML) (Xu et al., 2014).

In AML, CDK1 expression in BM from newly diagnosed patients was significantly lower than in BM from the same patients at AML relapse (Hedblom et al., 2013). However, there were no significant differences in CDK1 expression between BM of patients at diagnosis and that at remission. This suggests that CDK1 expression is associated with disease progression (Hedblom et al., 2013). Patients whose AML has higher levels of nuclear CDK1 tends to have poorer clinical outcome compared with those with lower levels (Hedblom et al., 2013). CDK1 is also required to modulate the response to all-trans retinoic acid (ATRA) through RARγ. For example, RARγ interaction with ATRA regulates protein levels of CDK1 and its subcellular localization; in turn, CDK1 modulates the levels of p27^kip1^ and AKT phosphorylation in response to ATRA treatment. Therefore, CDK1 modulation may represent a novel therapeutic approach to overcome ATRA resistance (Hedblom et al., 2013).

RUNX1 (also called AML1), a target of CDK1, plays an essential role in normal hematopoiesis (Link et al., 2010). It regulates lineage-specific genes and it directly stimulates G1 to S cell cycle progression (Redondo et al., 1992; Nuchprayoon et al., 1994; Zhang et al., 1994; Elagib et al., 2003). In agreement, adult *Runx1* conditional knockout mice show expansion of myeloid cells, T-cell developmental abnormalities and thrombocytopenia (Growney et al., 2005). Inactivating RUNX1 mutations have been described in myeloid neoplasms including MDS and cytogenetically normal AML, thus RUNX1 can be considered a tumor suppressor in these malignancies (Harada and Harada, 2011; Schnittger et al., 2011). However, a certain level of RUNX1 activity was required for the growth and survival of AML1-ETO and MLL-AF9 AML cells and combined loss of *Runx1/Cbfb* inhibited leukemia development induced by MLL-AF9 (Goyama et al., 2013). MLL binds to RUNX1 abrogating its proteasome-dependent degradation (Huang et al., 2011). CDK2 and CDK1 phosphorylate RUNX1 on Ser276 and Ser303. These phosphorylations lead to its degradation during G2/M by Cdc20-APC (Biggs et al., 2006). However, phosphorylation of RUNX1 at S48 and S424 by CDK1/cyclin B or CDK6/cyclin D3 modify RUNX1 to strengthen its ability to activate transcription and to stimulate hematopoietic cell proliferation (Zhang et al., 2008). In this context CDK1 inhibition may provide a therapeutic benefit in MLL-rearranged leukemia through its ability to block RUNX1. However, due to the dual role played by CDK1 in regulating RUNX1 caution should be exercised when targeting CDK1, as tissue-specific responses to CDK1 modulation may be counter-therapeutic.

Mutations in *FLT3* contribute to the development of AML as they cause self-phosphorylation and ligand-independent activation of the FLT3 receptor and downstream signaling pathways such as STAT3, STAT5, ERK1/2, and AKT (Hayakawa et al., 2000; Mizuki et al., 2000, 2003; Tse et al., 2000; Spiekermann et al., 2003). One of the targets of the ERK1/2 kinase is C/EBPα, a transcription factor playing a critical role in granulocytic differentiation (Ross et al., 2004). C/EBPα is often inactivated in various subtypes of leukemia by multiple mechanisms, including phosphorylation of serine 21 that inhibits its function and results in a differentiation block in FLT3-ITD leukemic blasts (Ross et al., 2004; Radomska et al., 2006). CDK1 is an FLT3-ITD-activated kinase, and is responsible for C/EBPα phosphorylation on serine 21 and for the blocking of its function (Radomska et al., 2012). Hence pharmacologic inhibitors of CDK1 may be useful for testing in *FLT3-ITD* driven leukemia.

Mutations of *EZH2, TET2, IDH1/2*, and *DNMT3A* involved in epigenetic regulation are frequently observed in adult AML, MDS, and myeloproliferative disease (MPD). Some of these gene mutations cause genome-wide aberration of epigenetic modifications (Sasaki et al., 2012) and have been proposed to play a role as disease drivers (Ding et al., 2012; Wakita et al., 2013). In CMML, the DNMT3A-R882H mutation confers a growth advantage of HSPCs. The transformational ability of this mutation operates through different mechanisms, including forming a protein complex with CDK1, resulting in enhanced cell cycle activity. Thus, CDK1 inhibition may be another approach to consider testing in CMML (Xu et al., 2014).

### CDK inhibitors targeting CDK1

Among CDK inhibitors, highly potent and selective pharmacological compounds targeting CDK1 have been more difficult to develop, probably because the crystal structure of CDK1 has not been reported, while the detailed structures of CDK2, 4 and 6 have been resolved (Wang et al., 2011). Moreover, the functions of CDK1/cyclin B1 in human cancer had not been properly elucidated similar to CDK4/CDK6/cyclin D. Multi-CDK inhibitors that target CDK1 are well-tolerated in cancer patients (Byrd et al., 2007; Tibes et al., 2008). In this section we will discuss selected pan CDK inhibitors that also target CDK1 and are used in clinical trials in hematologic malignancies.

### Flavopiridol

Flavopiridol (Alvocidib, Sanofi Aventis) is one of the first-generation CDK inhibitors. It is a pan-CDK inhibitor that inhibits CDK1, 2, 4, 6, 7, and 9 as well as GSK3β (Supplementary Table 4). Flavopiridol inhibits the C-terminal domain kinase activity of P-TEFb (CDK9/cyclin T) (IC_50_ 3 nM) (Chao et al., 2000) more potently than other CDKs including CDK7 (IC_50_ 110–300 nM) (Sedlacek, 2001). Furthermore, inhibition of CDK9 by flavopiridol is non-competitive with respect to ATP (Chao and Price, 2001). As a consequence of CDK9 inhibition, p-TEFb is inactivated and most RNA transcription *in vivo* is blocked that involves cell cycle and apoptosis regulators with short-lived mRNAs (Chao and Price, 2001; Lam et al., 2001).

Preclinical studies demonstrated that flavopiridol induces cell cycle arrest and apoptosis in hematopoietic cell lines of B-cell, T-cell and myeloid lineages (Parker et al., 1998). In preclinical animal models of localized and disseminated human leukemia and lymphoma, flavopiridol produces selective apoptosis of cells in the thymus, spleen, and lymph nodes, resulting in atrophy of these organs and induces complete or major regression of xenografts of human hematopoietic tumors (Arguello et al., 1998).

Flavopiridol was the first CDKI inhibitor to enter clinical trials in humans in the late 1990s (Senderowicz et al., 1998; Kelland, 2000). However, despite the promising preclinical findings, results of early clinical trials using flavopiridol as single agent employing either 72-h continuous or 1-h bolus infusion schedules were disappointing (Byrd et al., 2005). The use of flavopiridol in the clinic has also been hampered by complex pharmacokinetics requiring prolonged intravenous administration, and toxicities, including neutropenia, hypotension, and diarrhea (Dickson, 2014; Pitts et al., 2014).

Approximately 60 clinical trials using flavopiridol as a single agent or in combination with conventional chemotherapeutic drugs have been completed and/or are ongoing. Twenty six of these trials are for hematologic malignancies, including CLL, AML, ALL, CML, lymphocytic lymphoma, MCL, MM, and the remaining trials are for solid tumors, including breast, prostate, melanoma, pancreatic cancer, germ cell tumors, ovarian, lung, and esophageal cancer (clinicaltrials.gov accessed 11/2014). Most of these trials are completed or terminated. Only the currently active trials in hematologic malignancies are shown in (Supplementary Table 4).

Flavopiridol as 30-min bolus dose followed by a 4-h infusion produced sustained micromolar concentrations of flavopiridol for several hours in a phase I trial in patients with relapsed/refractory, genetically high-risk CLL; 45% PR was observed (Byrd et al., 2007). Hematologic improvement has been reported in a patient with hairy cell leukemia refractory to pentostatin and rituximab (Jones et al., 2012). When flavopiridol is given in a timed sequential fashion with cytarabine and mitoxantrone (FLAM regimen), direct anti-leukemia cytotoxicity and encouraging clinical responses are observed in patients with poor-risk newly diagnosed and relapsed/refractory AML. *FLT3*-mutant AMLs are particularly susceptible to the FLAM strategy (Karp et al., 2003, 2005, 2007, 2010, 2012). A phase 1 trial in 55 adults with relapsed/refractory acute leukemias began at a total flavopiridol dose of 50 mg/m^2^ per day 3 times (20 mg/m^2^ bolus, 30 mg/m^2^ infusion). DLTs occurred at level 6 (30 mg/m^2^ bolus, 70 mg/m^2^ infusion) with tumor lysis, hyperbilirubinemia, and mucositis. Death occurred in 5 patients (9%). CR occurred in 22 (40%) across all doses. Overall and disease-free survivals for CR patients were more than 60% at more than 2 years (Karp et al., 2011).

### Dinaciclib

Dinaciclib (SCH 727965, Merck) is a pyrazolo [1,5-α]pyrimidine that potently inhibits CDK1, CDK2, CDK5, and CDK9 (Parry et al., 2010; Paruch et al., 2010) with IC_50_ values ranging from 1 to 4 nM in a broad spectrum of human cancer cell lines including MCL, NHL, leukemia, colon, pancreatic, breast, prostate, SCLC, liver, and bladder (Parry et al., 2010). In addition to its direct interaction with the ATP-binding site, dinaciclib has also been shown to interact with the acetyl-lysine recognition site of bromodomains (Martin et al., 2013). In the preclinical testing dinaciclib demonstrated superior efficacy and therapeutic index compared to flavopiridol using the A2780 ovarian carcinoma murine xenograft model (Parry et al., 2010). The efficacy here refers to IC_50_ values and the minimal effective dose (MED) of both drugs given by the same schedule, associated with >50% tumor growth inhibition (Parry et al., 2010). Dinaciclib potently inhibits proliferation and induces apoptosis in a number of adult cancer cell lines and induces growth inhibition or regression in xenograft models using a variety of administration schedules (Parry et al., 2010). Preclinical evaluation of dinaciclib by the Pediatric Preclinical Testing Program (PPTP) in a panel of 23 cell lines showed a median IC_50_ of 7.5 nM (Gorlick et al., 2012). In an *in vivo* panel of 43 xenograft models of common childhood malignancies dinaciclib at a dose of 40 mg/kg administered intraperitoneally twice weekly for 2 weeks and repeated at day 21 with a total observation period of 6 weeks induced significant delays in event free survival distribution compared to control in 64% of solid tumor xenografts and in 43% of ALL xenografts (Gorlick et al., 2012). In patient-derived CLL cell lines, dinaciclib promotes apoptosis and abrogates microenvironment cytokine protection (Johnson et al., 2012). Dinaciclib inhibits Mcl-1 expression and induces PARP cleavage in peripheral blood mononuclear cells (PBMCs) from patients with AML and ALL 4 h after infusion. Prolonged exposures to dinaciclib, at clinically attainable concentrations, resulted in enhanced leukemia cell death (Gojo et al., 2013).

There are currently 16 clinical trials testing dinaciclib (SCH 727965) as single agent or in combination with other agents in patients with solid tumors (7 trials) and hematologic malignancies (9 trials) (clinicaltrials.gov accessed 11/2014). Of the nine trials for hematological malignancies, 2 trials have been terminated for patients with MCL or B-Cell CLL (Study P04715) and for those with AML or ALL (P04717AM2). Four studies are completed: Phase 1 Weekly Dosing of SCH 727965 in patients With advanced cancer (Study P04629AM6), including NHL, MM, B-cell CLL; Phase 1 Every-3-Week Dosing of SCH 727965 in Patients With Advanced Cancer (Study P04630) including NHL and MM; a third study in MM; and A Study of Dinaciclib in Combination With Rituximab in Participants With CLL and SLL (P07974). Three studies are currently active; one in MM, and two studied in CLL and lymphoma (Supplementary Table 4).

In a phase II study in adult patients with relapsed/refractory AML (*n* = 14) and ALL (*n* = 6) treated with dinaciclib at 50 mg/m^2^ given as a 2-h infusion every 21 days, most patients had dramatic but transient reduction in circulating blasts; however, no BM remissions were achieved on this schedule (Gojo et al., 2013). The most common toxicities were gastrointestinal, fatigue, transaminitis, and tumor lysis syndrome (TLS), including one patient who died of acute renal failure (Gojo et al., 2013). Based on the data that the expression of CDK5 is higher in MM compared to normal organs, a phase I/II trial of dinaciclib as a single agent was conducted in patients with relapsed MM at doses of 30–50 mg/m^2^, administered on day 1 of a 21-day cycle (Kumar et al., 2015). The MTD was determined to be 50 mg/m^2^ per day, the dose used in the Phase II portion (Bose et al., 2013). Overall, 27 evaluable patients were accrued; median number of prior therapies was 4. The overall confirmed response rate (≥PR) was 3 of 27 (11%); including one patient at 30-mg/m^2^ dose [1 very good response rate (VGPR)] and two patients at 40 mg/m^2^ dose (1 VGPR, 1 PR)] (Kumar et al., 2015). Leukopenia, thrombocytopenia, gastrointestinal symptoms, alopecia, and fatigue, were the most common adverse events. Because CDK5 inhibition can enhance the activity of proteasome inhibitors *in vitro*, ongoing studies are investigating the combination of dinaciclib with proteasome inhibitors (Kumar et al., 2015).

In a phase I study of dinaciclib in combination with rituximab in patients with relapsed/refractory CLL, only five patients completed the study due to early termination of the enrollment in the study and all presented with drug-related adverse events (AEs), but no DLTs were observed. The most commonly observed toxicities included hematological with a frequency of ≥40%, digestive and metabolic. Four patients achieved stable disease, and one patient achieved a CR. PK samples showed no obvious interaction between dinaciclib and rituximab (Fabre et al., 2014).

### AT7519

The pyrazole-3-carboxamide AT7519 (ASTEX) is another pan-CDK inhibitor that is highly active against CDK9 (IC_50_ <10 nM) and CDK5 (IC_50_ = 13 nM) followed by CDK2 (IC_50_ = 47 nM). It inhibits CDK1/cyclin B with an IC_50_ = 210 nM) (Squires et al., 2009) (Supplementary Table 4). Because of its high affinity toward CDK9, the mechanism of AT7519 is mainly through inhibition of transcription. Indeed, AT7519 inhibited RNA pol II phosphorylation, a CDK9 substrate, associated with decreased RNA synthesis in MM cell lines (Santo et al., 2010). Preclinical testing of AT7519 showed that it has antiproliferative activity and induces apoptosis in MM cell lines and MM tumor model through GSK3β activation and RNA pol II inhibition (Santo et al., 2010). AT7519 induces apoptosis at concentrations of 100 to 700 nM in leukemia cell lines and samples from patients with CLL with inhibition of phosphorylation of the RNA pol II, downregulation of Mcl-1, and PARP cleavage (Squires et al., 2010).

Currently there are four clinical trials with AT7519 as single agent or in combination with other agents (clinicaltrials.gov accessed 11/2014). Three are active trials: Phase I/II study in combination with bortezomib in MM, a Phase II study in MCL and a Phase II in CLL (Supplementary Table 4). One Phase I study has been completed in advanced or metastatic solid tumors or refractory NHL. AT7519 was given as a 1-h intravenous infusion on days 1, 4, 8, and 11 every 3 weeks. DLTs included mucositis, febrile neutropenia, rash, fatigue and hypokalemia. The recommended phase II dose (RP2D) was 27.0 mg/m^2^ per day. Ten of 19 patients had stable disease as the best response (median duration: 3.3 months; range: 2.5–11.1 months) (Chen et al., 2014).

### P276-00

P276-00 (Nicholas Piramal, India), a flavone derivative, is an ATP competitive small molecule inhibitor of CDK9 (IC_50_ = 63 nM), CDK4 (IC_50_ = 79 nM) and CDK1 (IC_50_ = 224 nM). P276-00 shows potent antiproliferative effects against various human cancer cell lines including the AML cell line (HL-60) and is highly selective for cancer cells as compared with normal fibroblast cells (Joshi et al., 2007). P276-00 decreases cellular levels of cyclin D1, an effect that can be attributed to inhibition of CDK9 transcriptional activity. It inhibits CDK4/cyclin D1 activity, downregulates pRb Ser^780^ phosphorylation, and induces apoptosis by the induction of caspase-3 (Joshi et al., 2007). P276-00 also downregulates Mcl-1 and induces PARP cleavage in MM cell lines (Raje et al., 2009) through inhibition of CDK9-T1 and RNA pol II-dependent transcription (Manohar et al., 2011) and shows antitumor activity in a MM xenograft model. Combination with bortezomib results in synergism (Raje et al., 2009). Similar results were obtained in MCL *in vitro* and *in vivo* (Shirsath et al., 2012). Synergistic effects with doxorubicin at suboptimal doses in NSCLC cell lines are also reported (Rathos et al., 2013).

There are seven completed and three terminated Phase I or Phase II studies. One trial is in MM, another Phase II study includes patients with MCL and the remaining studies are in solid tumors (clinicaltrials.gov, accessed 11/2014) (Supplementary Table 4).

### TG02

TG02 (Tragara Pharmaceuticals) is a pyrimidine-based pan-CDK/FLT3/JAK2 inhibitor that inhibits CDKs 1, 2, 7, and 9 together with JAK2, FLT3 (William et al., 2012), and ERK5 (Alvarez-Fernandez et al., 2013; Ortiz-Ruiz et al., 2014) with IC_50_ of 13, 73, and 56 nM for CDK2, JAK2, and FLT3, respectively (William et al., 2012). TG02 is anti-proliferative in a broad range of tumor cell lines, in primary cultures of progenitor cells derived from AML and from patients with *polycythemia ver*a, inducing G1 cell cycle arrest and apoptosis. *In vivo*, TG02 induces tumor regression after oral dosing on both daily and intermittent schedules in a murine model of FLT3-ITD leukemia (MV4-11) and prolongs survival in a disseminated AML model with wt FLT3 and JAK2 expressed in HL-60 AML cell lines (Goh et al., 2012). TG02 at 100 nM is potently cytotoxic toward CD34(+) CD38(−) CD123(+) positive and unfractionated AML cells from patients. The antitumor activity of TG02 is likely mediated by dephosphorylation of RNA Poly II leading to transcription repression of survival molecules such as Mcl-1 and XIAP, with subsequent BAX activation and apoptosis (Pallis et al., 2012; Ortiz-Ruiz et al., 2014). TG02 induced similar effects in MM cell lines, displayed significant activity in two MM xenograft models, and enhanced the *in vivo* activity of bortezomib and lenalidomide (Alvarez-Fernandez et al., 2013; Ortiz-Ruiz et al., 2014) in a xenograft model of triple negative breast cancer (Ortiz-Ruiz et al., 2014).

Currently there are two active Phase I clinical trials of TG02 in CLL, small lymphocytic lymphoma and in advanced hematologic malignancies. The latter consists of three arms (1) TG02 as single agent in patients with acute leukemia, (2) as single agent in patients with MM and (3) TG02 in combination with carfilzomib in patients with MM (Supplementary Table 4) (clinical trials.gov accessed 11/2014).

### RGB-286638

RBG-286638 (Agennix) is a novel indenopyrazole-derivative, multi-kinase inhibitor. It potently inhibits CDKs 1, 2, 3, 4, 5, 6, 7, 9 as well as non-CDK kinases such as Jak1, Jak2, c-Src, AMPK, and GSK3β (Supplementary Table 4) (Cirstea et al., 2013). RGB-286638 is cytotoxic *in vitro* in MM cell lines with either wild type or mutant p53 and inhibits MM tumor growth and prolongs survival *in vivo*. It induces caspase-dependent apoptosis regardless of p53 status. It acts *via* down regulation of RNA pol II phosphorylation and inhibition of transcription. RGB-286638 also down regulates the oncogenic miR-19, miR-92a-1, and miR-21 (Cirstea et al., 2013). So far only one Phase I study of RGB-286638 in patients with solid tumors has been conducted. RGB-286638 was given intravenously on days 1–5 every 28 days. Twenty-six patients were enrolled at 6-dose levels from 10 to 160 mg/d. Four DLTs were observed in two of the six patients enrolled at the highest dose level. These toxicities were AST/ALT elevations in one patient, paroxysmal supraventricular tachycardias (SVTs), hypotension, and an increase in troponin T. The recommended MTD for phase II studies is 120 mg/d. Prolonged disease stabilization (range, 2–14 months) was seen across different dose levels (Van Der Biessen et al., 2014).

### Teramperprocol

Terameprocol (formerly known as EM-1421 and M4N) (Erimos Pharmaceuticals) is a semisynthetic derivative of a naturally occurring plant lignan. It selectively inhibits specificity protein 1 (Sp1)-regulated proteins, including CDK1, survivin and VEGF (Castro-Gamero et al., 2013), thus inhibiting the cell cycle, triggering apoptosis and decreasing angiogenesis. Preclinical studies have demonstrated the potent anticancer activity of terameprocol in tumor cell lines and animal models (Smolewski, 2008).

There have been 6 clinical trials with terameprocol (EM-1421) (clinicaltrials.gov, accessed 11/2014). In a phase I clinical trial including 25 patients with solid tumors, 8 patients exhibited stable disease and 17 had progressive disease; the drug was generally well-tolerated (Smolewski, 2008). Other phase I/II clinical trials have been for the treatment of glioma, treatment-refractory solid tumors, and cervical dysplasia. A Phase I study in patients with advanced leukemias was conducted with 16 patients treated with 1000, 1500, or 2200 mg of intravenous terameprocol 3×/week for 2 of 3 weeks. No DLT was observed, and the RP2D was determined to be 1500 mg 3×/week for 2 of 3 weeks cycle. Five AML patients had stable disease greater/equal to 2 months (Tibes et al., 2014).

### WEE-1 kinase

WEE-1 is a protein kinase that plays a central role in the proper timing of cell division cycle by modulating the activities of CDK1 and CDK2 through inhibitory phosphorylation of conserved Tyr15 residues on both kinases, thereby controlling entry into mitosis and DNA replication during S phase (Do et al., 2013). WEE-1 plays a critical role in maintaining genome integrity and is essential for embryonic survival at the pre-implantation stage of mouse development (Tominaga et al., 2006). Mice deficient for WEE-1 die before E3.5. *Wee1*^−/−^ embryos are defective in the G2/M cell cycle checkpoint induced by γ-irradiation resulting in widespread apoptosis. Acute ablation of WEE-1 in MEFs results in growth defects and cell death, aneuploidy, DNA damage, and CHK2 activation (Tominaga et al., 2006).

**Figure 2:**
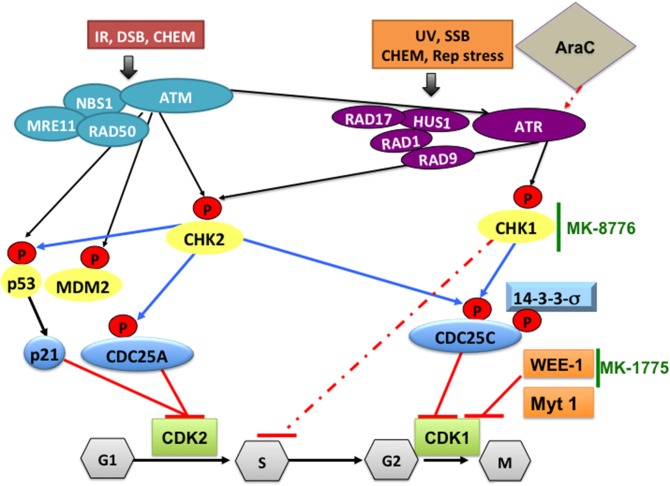
**DNA damage pathways and how CHK1-, WEE1-, or CDK1 inhibitors can synergize with cytarabine to increase its DNA damaging efficacy in AML**. Ataxia-telangectasia mutated (ATM) protein kinase is activated in response to ionizing radiation (IR), radiomimetic agents, and agents which cause double-strand DNA breaks (DSBs). Ataxia-telangiectasia-related (ATR) protein kinase is activated by a broader range of genotoxic stimuli that result in single-strand DNA breaks (SSB). In addition, ATM can also activate ATR. Initial sensing of DNA damage can also be mediated by the NBS1-MRE11-RAD50 complex and by RAD17-RAD1-HUS1-RAD9 at the site of DNA breaks. This is followed by phosphorylating CHK2, p53, MDM2 and CHK1, which mediate cell cycle checkpoint responses to arrest the cells until DNA damage is repaired. Activated p53 will induce p21 and the CDC25A protein, which inhibit CDK2 and prevents G1/S transition. Altered phosphorylation (by CHK1) or cytoplasmic sequestration of CDC25C (by 14-3-3σ) will inactivate CDK1/cyclin B preventing cells from entering mitosis. CHK1 also phosphorylates/activates WEE-1, which phosphorylates and inactivates CDK1/cyclin B complex on Tyr15 residue, resulting in cell cycle arrest at G2, allowing time for DNA repair. In AML, treatment with cytarabine (Ara-C) activates the DNA damage pathway through CHK1, which stabilizes stalled replication fork and induces S phase arrest. This will allow DNA damage induced by Ara-C to be repaired. To increase the DNA damaging efficacy of Ara-C the checkpoint pathway can be blocked by a selective CHK1 inhibitor such as MK-8776 that overrides the S-phase checkpoint activation. To overcome the G2/M checkpoint blocking WEE-1 kinase using the selective inhibitor MK-1775 will result in CDK1 activation and cells die due to accumulation of genetic lesions. CHEM, genotoxic chemicals; Rep stress, replication stress; UV, ultra violet radiation.

In the mammalian cell cycle there are three checkpoints that function in G1, S and G2, respectively. Depending on the type of genotoxic stress, either ataxia-telangiectasia mutated (ATM) protein kinase (Supplementary Table 2, Figure 2) or ataxia-telangiectasia-related (ATR) protein kinase is preferentially activated (Do et al., 2013). ATM is activated in response to ionizing radiation, radiomimetic agents, and agents, which cause double-strand DNA breaks (DSBs). It phosphorylates and activates CHK2, which, in turn, phosphorylates CDC25C at Ser216, creating a binding site for the 14-3-3σ protein (Matsuoka et al., 2000). This leads to nuclear export and cytoplasmic sequestration of CDC25C. Suppression of CDC25C phosphatase activity results in inhibitory phosphorylation of the CDK1/cyclin B complex, maintaining CDK1 in an inactive form and preventing entry into mitosis (Figure 2). If the checkpoints are defective (or pharmacologically inhibited) DNA damage will not be repaired and cells die due to accumulation of genetic lesions. ATR is activated by a broader range of genotoxic stimuli that result in single-strand DNA breaks (Jazayeri et al., 2006; Johnson et al., 2009). ATR phosphorylates and activates CHK1 that can also be activated by ATM. CHK1 then phosphorylates WEE-1 and CDC25C, thereby activating WEE-1 kinase activity and inactivating CDC25C phosphatase activity. WEE-1, then, phosphorylates and inactivates CDK1/cyclin B complex on Tyr15 residue, resulting in cell cycle arrest at G2, allowing time for DNA repair (Do et al., 2013).

The concept of using WEE-1 inhibitors in cancer therapy is based on the notion that by abrogating the G2 checkpoint, in the presence of DNA damaging agents such as cytarabine, used in the treatment of AML, cells would die in mitosis (mitotic catastrophe). This would be of particular relevance in cells in which p53 is deleted or mutated, as is the case in many human cancer types. The G1 checkpoint is controlled by p53 (Figure 2). If p53 is defective, genetic lesions will accumulate and cells will depend on the G2 checkpoint for repair, but if the G2 checkpoint is also non-functional, therefore, unrepaired DNA will further accumulate leading to mitotic catastrophe and/or death by apoptosis. Indeed, the combination of DNA-damaging cancer therapy with WEE-1 inhibition catalyzes mitotic catastrophe (De Witt Hamer et al., 2011). The WEE-1 inhibitor PD0166285 abrogates G2/M checkpoint inducing early cell division in the B16 melanoma cell line and this effect is related to microtubule stabilization and decrease in cyclin D transcription (Hashimoto et al., 2006). Using a microtubule interfering compound we observed a similar phenotype of accumulation of cells in mitosis with high CDK1 activity that leads to mitotic catastrophe and apoptosis (Waraky et al., 2014).

Checkpoint kinases (CHK1 and CHK2) are also considered therapeutic targets in cancer including hematologic malignancies. The therapeutic targeting of checkpoint kinases and mitotic kinases (Polo-like kinases and Aurora kinases) were previously discussed in details in recent reviews (Lapenna and Giordano, 2009; Moore et al., 2013; Gojo and Karp, 2014; Pitts et al., 2014).

### WEE-1 in human cancer

WEE-1 is overexpressed in several cancers including primary AML, ALL, and CML specimens (Tibes et al., 2012). High expression of WEE-1 in malignant melanoma correlates with poor disease-free survival (Magnussen et al., 2012). Recently, an INDEL was identified in the poly-T track of the 56 bp non-coding region of WEE-1 in pancreatic adenocarcinoma, which contains the binding site of an RNA binding protein, HuR (important for cancer cell survival). The incidence of a TT insertion is increased in familial pancreatic cancer and results in decreased WEE-1 expression upon DNA damage (Lal et al., 2014). This INDEL can have clinical implications in predicting chemo-responsiveness. WEE-1 was also identified as a therapeutic target in AML (Porter et al., 2012a), in medulloblastoma (Harris et al., 2014)and in head and neck cancer (Wu et al., 2011). Inhibition of WEE-1 either by PD0166285 or by siRNA knockdown sensitizes solid tumor cell lines to DNA damage by irradiation and topoisomerase inhibition (Wang et al., 2001, 2004; Posthumadeboer et al., 2011).

## Pharmacological inhibitor of WEE-1 kinase

### MK-1775

MK-1775 (AZD1775, Merck), a recently developed pyrazolo-pyrimidine derivative is a potent and selective ATP-competitive small-molecule inhibitor of WEE-1 kinase with an IC_50_ value of 5.2 nM in *in vitro* kinase assays.

The WEE-1 inhibitor MK-1775 shares common mechanisms of cell death with CHK1 inhibitors and combined WEE-1 and CHK1 inhibition forces mitotic entry from S-phase in the absence of chemotherapy (also achieved by WEE-1 inhibition alone). p53/p21 inactivation combined with high expression of mitotic cyclins and EZH2 predispose cells to mitotic entry during S-phase (Aarts et al., 2012). Combined treatment of a CHK1 inhibitor (PF-00477736) and MK-1775 confirmed the marked synergistic effect in various human cancer cell lines (breast, ovarian, colon, prostate), independently of the p53 status. Cancer cells undergo premature mitosis before the end of DNA replication, with damaged DNA leading to cell death partly by apoptosis. These effects translated into increased tumor growth inhibition in a xenograft model of ovarian cancer than with each inhibitor as single agent and with no toxicity (Carrassa et al., 2012).

### Combination of MK-1775 with other agents enhances therapeutic effects in preclinical models of leukemia

WEE-1 was identified as a therapeutic target for mutant RAS-driven AML cell lines and primary patient samples using a chemical screen to identify compounds capable of potentiating mammalian target of rapamycin (mTOR) inhibition. The observed synergy enhances dephosphorylation of AKT, 4E-binding protein 1 and s6 kinase, and correlates with increased apoptosis. These findings suggest a combined WEE-1 /mTOR inhibition as a novel therapeutic strategy for the selective treatment of mutant *RAS*-positive leukemia (Weisberg et al., 2015). Using RNAi screening of the kinome with cytarabine and a genome-wide shRNA screen, WEE-1 was also identified as a therapeutic target in myeloid leukemia (Tibes et al., 2012) and a critical mediator of AML cell survival after cytarabine exposure (Porter et al., 2012a). Pharmacologic inhibition of WEE-1 is synergistic with cytarabine (Figure 2), increases apoptosis more than either drug alone, and prevents S-phase arrest induced by cytarabine in AML cell lines and primary cells (Porter et al., 2012a), as well as in ALL and CML cell lines (Tibes et al., 2012). Similar observations have been reported in pediatric Down Syndrome AML (DS-AML) cell lines CMK and CMY, as well as primary DS-AML patient samples (Caldwell et al., 2014). Adding MK1775 to cytarabine substantially reduced viability in 13 of 14 AML, CML, and MDS patient samples compared with cytarabine alone (Caldwell et al., 2014).

Furthermore, MK-1775 produces synergistic inhibition in combination with histone deacetylase inhibitors (HDACIs, for example, vorinostat), which interrupt the DNA damage response, to kill p53- wt or—mutant leukemia (Zhou et al., 2014). In addition, a similar effect has been observed in FLT3-ITD leukemia cells in association with pronounced WEE-1 inhibition and diminished CDK1 Tyr15 phosphorylation (Zhou et al., 2014). The combination is active against patient-derived AML cells harboring either wt or mutant p53. CD34+/CD123+/CD38- populations enriched for leukemia-initiating progenitors, but not CD34+ normal hematopoietic progenitors, are highly susceptible to this regimen. The combination of MK-1775 with vorinostat in AML murine xenografts significantly reduces tumor burden and prolongs animal survival (Zhou et al., 2014). Therefore, in AML, mutant p53 is not a predictive biomarker for response to WEE-1 inhibition. This result was also confirmed in another preclinical study after combination of cytarabine and MK-1775 for the treatment of AML cell lines and AML mouse model (Van Linden et al., 2013). Pre-treatment of the human leukemic T-cell lines, Jurkat and MOLT-4, with the WEE-1 inhibitor 681641 or the Rad51 inhibitor RI-1 increases the sensitivity of Jurkat cells to irradiation. However, combining both inhibitors together results in a further enhancement of apoptosis (Havelek et al., 2014).

Similar to solid tumors, AML cell lines and patients' blast samples are sensitive to MK-1775 and to the combined effect of MK-1775 and a CHK1 inhibitor. Patient samples harboring t(15;17) translocation are significantly more sensitive to MK-1775 than non-t(15;17). CHK1 induction by MK-1775 is suggested as a potential mechanism of resistance to MK-1775 treatment. MK-1775 induces DNA damage, which activates CHK1. CHK1 phosphorylates CDC25, inhibiting the dephosphorylation of CDK1/2, thus countering the effects of MK-1775. Therefore, activation of CHK1 can be overcome by the addition of a CHK1 inhibitor, resulting in a synergistic, anti-leukemic activity (Qi et al., 2014).

There are 20 clinical studies with MK-1775 in solid tumors in Phase I and II stages, but no trials in hematological malignancies yet (Supplementary Table 4) (clinicaltrials.gov, accessed 11/2014).

### Cyclin C-CDK3/CDK8/CDK19

Cyclin C associates with CDK3 to phosphorylate pRB, thus promoting cell cycle entry from quiescence (Ren and Rollins, 2004). Indeed, knockdown of cyclin C increases quiescence in HSPC (Miyata et al., 2010). Cyclin C also associates with CDK8 (Leclerc et al., 1996) and with CDK8-related CDK19 (Tassan et al., 1995; Li et al., 2014). This complex has a major role in transcription. It is a component of RNA pol II transcription initiation complexes and represses transcription by phosphorylating the CTD of the largest RNA pol II subunit (Liao et al., 1995; Tassan et al., 1995; Leclerc et al., 1996; Rickert et al., 1996), as well as by inhibiting the general transcription factor TFIIH (Akoulitchev et al., 2000). Cyclin C/CDK8 is also incorporated into the inhibitory module of the transcriptional mediator complex, and blocks the interaction of the mediator complex with RNA pol II (Elmlund et al., 2006; Knuesel et al., 2009). In fact, cyclin C/CDK8 plays a dual role in regulating transcription; as a negative regulator through phosphorylation and suppression of transcription factors (Chi et al., 2001; Nelson et al., 2003; Fryer et al., 2004; Alarcon et al., 2009), or by activating transcription either as a part of the basal transcriptional machinery, or downstream of p53, and of the Wnt/β-catenin pathway (Donner et al., 2007, 2010; Furumoto et al., 2007; Firestein et al., 2008; Morris et al., 2008).

### Mouse models reveal a critical role for cyclin C as a tumor suppressor in T-ALL

Genetic ablation of cyclin C in the mouse results in embryonic lethality at E10.5 (Li et al., 2014). Cyclin C plays a major role in hematopoiesis. Ablation of cyclin C in the hematopoietic system *in vivo* results in enlargement of the thymus and stabilization of the intracellular Notch1 (ICN1) proteins (Li et al., 2014). Indeed, ICN1 is phosphorylated by cyclin C/CDK8 complexes resulting in increased ubiquitination by the SCF (Skip1–Cullin1–F-box)–Fbw7 ubiquitin ligase that leads to proteasome-dependent degradation (Fryer et al., 2004; Thompson et al., 2007). Activation of Notch1 is triggered by a ligand-dependent proteolytic cleavage that results in the release of the ICN1 protein, which translocates to the nucleus, thereby inducing target-gene transcription (Gordon et al., 2008; Trakala and Malumbres, 2014). Notch1 is a potent T-cell oncogene, with >50% of T-ALL cases carry activating mutations in the Notch1 receptor, many of which truncate the Notch1 C-terminal domain containing the PEST domain, resulting in increased ICN1 stability. Cyclin C/CDK3/CDK8/CDK19 complexes phosphorylate ICN1 on residues T2512, S2514, and S2517 located in the PEST-domain leading to its degradation (Li et al., 2014). Therefore, cyclin C/CDK3/CDK8/CDK19 complexes function as tumor suppressors in T-ALL by preventing the accumulation of ICN1 and the lack of these complexes led to enhanced T-ALL development in Lck-LMO1 transgenic mice that mimic the alterations found in human T-ALL (Li et al., 2014). It is noteworthy to mention that Notch appears to have different effects in hematologic malignancies. While Notch signaling inhibits AML growth and survival, thus functioning as a tumor suppressor in AML (Kannan et al., 2013), in other studies Notch signaling had variable effects on AML growth and survival, depending on the individual AML sample (Tohda et al., 2005).

### Cyclin C/CDK8-CDK19 in human cancer

In human, *CCNC* and *CDK19* genes lie very close to each other on the 6q21 region of the human chromosome 6, a region that is frequently deleted in multiple malignancies including breast, lung, ovarian, prostate carcinomas, leukemia and osteosarcoma (Trakala and Malumbres, 2014). Co-deletion of *CCNC* and *CDK19* takes place in 10% of T-ALL (Li et al., 2014) or 7% of DLBCL (Trakala and Malumbres, 2014). In addition, five mutations (out of 73 T-ALL patients) were identified in the cyclin-C/CDK recognition sites in the Cdc4 phosphodegron of Notch1 leading to ICN1 stabilization and were able to enhance T-ALL development when reproduced in mouse models, supporting the notion that alteration of the cyclin-C/CDK-dependent regulation of Notch1 contributes to human leukemia (Li et al., 2014). While *CDK19* is downregulated in leukemia, lymphoma and esophageal cancer, *CDK8* is overexpressed and amplified in ~50% of colon cancers suggesting a selective role of CDK8 in this type of tumors (Firestein et al., 2008; Morris et al., 2008). CDK8 phosphorylates and inhibits E2F1, an inhibitor of β-catenin function, resulting in increased activity of the Wnt/β-catenin pathway in colon cancer cells (Morris et al., 2008) and suppression of CDK8 expression in these cells inhibits proliferation (Firestein et al., 2008). CDK8 and cyclin C expression also correlate with poor survival in breast cancer and with platinum treatment failure in ovarian cancer (Porter et al., 2012b). Therefore, because of the dual role of the cyclin C/CDK8/CDK19 complexes functioning in some tumors as oncogenes and in other tumors as tumor suppressor genes the use of pharmacological inhibitors of this complex should be carefully considered depending on tissue type.

### Pharmacological inhibitor of CDK8

Senexin A was reported to bind CDK8 at the ATP pocket and to inhibit its cellular functions (IC_50_ = 280 nM) including the potentiation of β-catenin–dependent transcription. It also binds to CDK19 in an ATP-competitive manner (Porter et al., 2012b). In these studies p21 was found to bind to CDK8 and stimulate its kinase activity and was required for disease-promoting paracrine activities associated with DNA damage and senescence induced by chemotherapy. Senexin A also increases the efficacy of chemotherapy against xenografts formed by tumor cell/fibroblast mixtures (Porter et al., 2012b). It inhibits p21-induced transcription and reverses doxorubicin-induced tumor-promoting paracrine activities *in vivo* (Porter et al., 2012b).

Due to their potential ability to stabilize Notch, cyclin C/CDK8/19 inhibitors may be of therapeutic benefit for the hematologic malignancies in which Notch functions as a tumor suppressor such as CML (Yin et al., 2009; Yang et al., 2013b) and CMML (Klinakis et al., 2011).

## Concluding remarks

The main function of cell cycle protein kinases, including CDKs, was initially identified as drivers of cell cycle progression. In part because dysregulation of this process is one of the hallmarks of cancer, several first generation pharmacological inhibitors targeting CDKs have been developed. Most of these are ATP competitive inhibitors. However, major drawbacks of these pan CDK inhibitors include lack of selectivity toward tumor cells, off-target effects, which increase their toxicity, and unfavorable pharmacokinetics. In contrast, specific CDK inhibitors should provide selectivity and reduced toxicity because tumor cells may be selectively dependent on a certain pathway such as the cyclin D/CDK4/6/Rb pathway. The further development of specific CDK inhibitors may lead to significant advances in cancer treatment.

Experiments using mouse models deficient for CDKs have revealed that they also regulate differentiation, stem cell renewal, DNA repair, angiogenesis, and transcription with selective tissue dependence. Some CDKs act as oncogenes, while others function as tumor suppressors; they are altered in cancer by functionally relevant mutations and/or by altered expression and activities. Such information has led to a renewed interest in these pathways as therapeutic targets. For example, the CDK4/6 inhibitor, palbociclib, is emerging as a potentially useful agent after more than a decade in drug development limbo. Palbociclib and the related LEE011, as well as dinaciclib, are currently in Phase III clinical trials. Palbociclib has demonstrated promising single-agent activity in MCL.

Most of the pan-CDK inhibitors induce cell cycle arrest, apoptosis and/or transcriptional repression. Improved anti-tumor responses are usually observed when this class of molecularly targeted agents is used in combination with traditional cytotoxic drugs, such as flavopiridol and cytarabine for AML. Alternatively, combination with other targeted compounds, such as proteasome inhibitors (bortezomib) in MM or NHL has shown promising enhancement of anti-tumor responses. The disruption of DNA repair pathways using CHK1 inhibitors has shown synergy with cytarabine in preclinical studies of AML. Similar results have been observed with combinations of cytarabine and WEE-1 inhibitors. Another identified complex that plays a critical role in the development of Notch-dependent T-ALL is the cyclin C with its associated kinases CDK3/8/19. Further studies of this pathway in different types of human cancer and the development of compounds targeting these complexes are needed.

An unexplored yet important area that also needs to be addressed is the efficacy of cell cycle inhibitors in pediatric malignancies. Pediatric AML, for example, remains a challenging disease with overall survival between 50 and 60%. Conventional AML therapy is basically the same for adults and children, based on cytarabine and anthracycline combinations. Relapsed AML refractory to chemotherapy has an overall survival less than 20%. Cell cycle inhibitors are currently in clinical trials as targeted agents in pediatric AML or ALL; the Aurora A inhibitor alisertib in a phase II trial and the Aurora A/B inhibitor AT9283 is in phase I (Napper and Watson, 2013). The WEE-1 kinase inhibitor MK-1775 in combination with cytarabine may be another promising new treatment approach for Down Syndrome-AML that will need clinical testing.

A key question regarding the clinical development of cell cycle inhibitors in pediatric AML is the identification of the molecular signatures that predict susceptibility to these compounds. One approach is currently being tested, as part of a national St. Baldrick Foundation (SBF) Pediatric AML Consortium (SBF-PAC). This consortium is focused on the clinically real time identification of altered key leukemia pathways along with the integration of functional assays, such as kinase inhibition screens, single cell network profiling and drug inhibition screens, to define and validate driver pathways. This information is linked to a clinical trial designed to test molecularly directed therapies in pediatric AML. Some of the pathways identified define pivotal genes regulating leukemia survival, cell proliferation and pathways driving leukemia resistance to conventional therapies. In this context, further exploration of inhibitors targeting cell cycle kinases in pediatric AML is warranted.

### Conflict of interest statement

The authors declare that the research was conducted in the absence of any commercial or financial relationships that could be construed as a potential conflict of interest.
